# Supplementary material for: The Role of Amino Acid Metabolism of Tumor Associated Macrophages in the Development of Colorectal Cancer
Source: Cells. 2022 Dec 17;11(24):4106. doi: 10.3390/cells11244106 (PMC9776905; doi:10.3390/cells11244106)
Supplement: Supplementary file 1 [file cells-11-04106-s001.zip › cells-1948938-supplementary.pdf]

Method of primer design:

First, input the target gene in the GENE column of NCBI, and then select the mouse source to find the mRNA of the target gene and copy the longest mRNA sequence. Then enter Primer3 Input, copy the mRNA sequence into primer3, and click pick printers. The QPCR primer of the target gene can be preliminarily obtained. Next, copy the NM number of mRNA into the prime blast of NCBI; At the same time, copy the QPCR primer sequence obtained above into the primer blast, and finally click get primers to obtain the primer sequence.

Method of ELISA:

1. Coating process: Dilute the used antigen with coating diluent to an appropriate concentration, and add 100μl antigen into each hole. A blank control and a negative control shall be set, which shall be incubated at 37 ℃ for 4h, and the liquid in the hole shall be discarded.

2. Closure: 5% fetal bovine blood is kept at 37 ℃ for 40 min. Be careful not to have bubbles. After sealing, wash with wash buffer for 3 times, 3 minutes each time.

3. Add the sample to be tested: the sample is diluted at 1:50, and the diluted sample is added into the enzyme labeled reaction hole. Three replicates are set for each sample, 100μl for each hole. Incubate at 37 ℃ for 60min. Wash 3 times with wash buffer, 3min each time.

4. Add enzyme labeled antibody: use 1:100 dilution for enzyme labeled antibody, 100μl per hole. Incubate at 37 ℃ for 60min.Wash 3 times with wash buffer, 3min each time.

5. Add substrate solution 100μl per hole. Place it at 37 ℃ away from light for 5 min, and add stop solution for color development.

6. Termination reaction: Add 50μL terminating liquid into each hole. Stop the reaction and determine the experimental results within 20 minutes.
